# Impact of invasive weed *Parthenium hysterophorus* (Asteraceae) on mosquito abundance and plant-feeding behavior in an arboviral endemic region in Kenya

**DOI:** 10.1186/s13071-025-07174-3

**Published:** 2025-12-12

**Authors:** Tasneem Osman, Tatenda Chiuya, Eric M. Fèvre, Sandra Junglen, Christian Borgemeister

**Affiliations:** 1 Center for Development Research (ZEF), University of Bonn, Bonn, Germany; 2 International Livestock Research Institute (ILRI), Nairobi, Kenya; 3 Institute of Infection, Veterinary and Ecological Sciences, University of Liverpool, Liverpool, UK; 4 Institute of Virology, Charité–Universitätsmedizin Berlin, corporate member of Freie Universität Berlin, Humboldt-Universität zu Berlin and Berlin Institute of Health, Berlin, Germany

**Keywords:** *Parthenium hysterophorus*, Invasive plant, Mosquito, Ecosystem health

## Abstract

**Background:**

Invasive alien species (IAS) are rapidly altering ecosystems, undermining biodiversity, ecosystem processes, and interspecies interactions. Although IAS ecological and economic effects are well recognised, their impact on mosquito populations and the dynamics of infectious diseases is poorly understood. Plant-derived sugars are crucial for mosquito biology, supporting nectarivorous male survival and enhancing female blood feeding.

**Methods:**

In this study, we investigated how Parthenium hysterophorus, a rapidly proliferating invasive weed, shapes the population structure and nectar-feeding behaviour of the mosquito vector in the Rift Valley area of Kenya. Across six villages, three heavily infested with P. hysterophorus and three uninfested controls, we collected 48,489 mosquitoes representing 35 species from two subfamilies (Anophelinae and Culicinae) and nine genera, including Anopheles, Aedes, Culex, Mansonia, and Coquillettidia. Mosquito plant feeding was confirmed using the anthrone test, and the ingested flora were identified via DNA barcoding of chloroplast markers, specifically matK, rbcL, and ITS2.

**Result:**

Mosquito abundance was significantly higher in Parthenium-infested villages, particularly during the dry season (p < 0.001), despite similar species diversity across sites. Medically important vectors, including Mansonia africana, Coquillettidia metallicus, Culex pipiens, and Anopheles funestus, were notably more common in invaded habitats. Overall fructose positivity was significantly high in mosquitoes from Parthenium sites (p = 0.046), with females showing especially higher rates (28.1% vs 18.0%; p = 0.0038). DNA barcoding indicated a clear feeding preference for P. hysterophorus among Coq. metallicus, Mn. africana, and An. funestus, alongside other plants such as Lantana camara.

**Conclusion:**

Our findings indicate that P. hysterophorus has a notable impact on mosquito population composition and stimulates sugar-feeding behavior among important vector species. This IAS acts as a sustainable nutritional source, potentially enhancing mosquito survival, extending vector activity in dry seasons, and heightening the risk of arboviral disease transmission. The findings highlight the critical need to integrate invasive plant management within comprehensive mosquito control strategies.

**Graphical Abstract:**

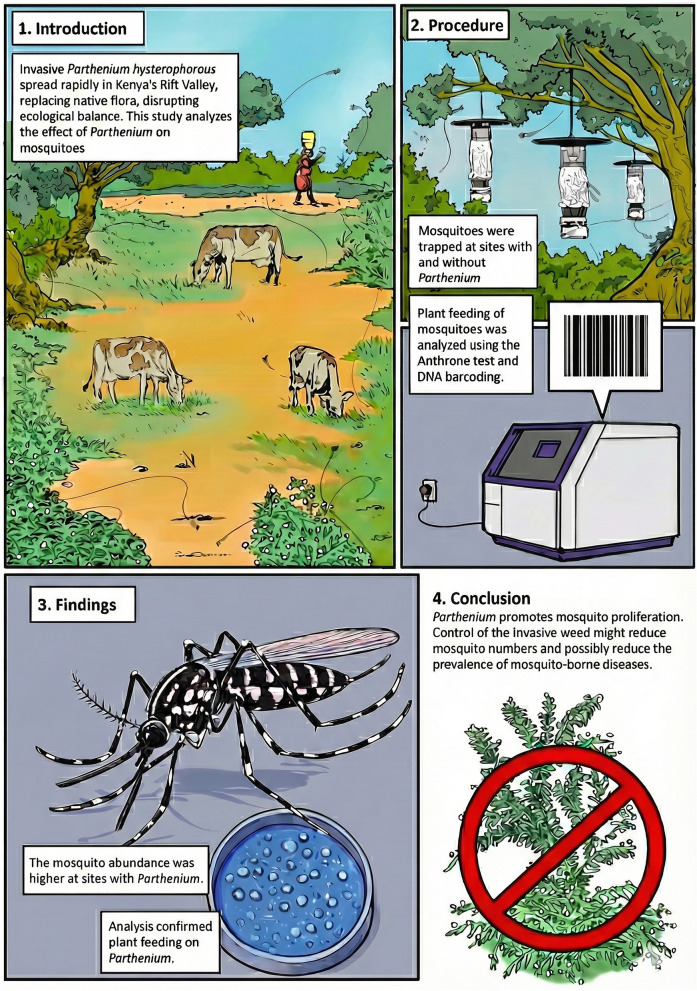

## Background

Invasive alien species (IAS) threaten the integrity of ecosystems throughout the world, affecting native species diversity and biological integrity [1]. Various factors, such as climate change, globalization, and reduced agricultural productivity, contribute to the spread and establishment of IAS [2]. With the increasing movement of people and goods around the world, fueled by new trade routes and enhanced transportation, the number of species accidentally introduced into new areas is rising constantly [3, 4]. Among the most aggressive and ecologically disruptive invasive species is *Parthenium hysterophorus* L. (Asteraceae). This rapidly expanding nonnative plant aggressively occupies regions outside its indigenous habitat in Central and South America, mainly Mexico and the Caribbean region, presenting significant risks to ecological diversity and farming practices, while also demonstrating harmful effects on both livestock and humans [5].

Mosquitoes depend on plant sugars as their primary source of energy. Males are exclusively herbivorous, while females enhance their blood meals with plant sugars to aid in flight, survival, and reproduction [6, 7]. The plant-feeding behavior of mosquitoes plays a crucial role in their biology, affecting their lifespan, reproductive success, and overall ability to transmit diseases. Nectar-rich plants can enhance mosquito populations, thereby facilitating the transmission of mosquito-borne diseases [8]. Mosquitoes demonstrate a distinct and selective preference for specific plant species while seeking sugar sources, and these preferred plants play a crucial role in mosquito survival by supplying essential sugar sources that boost energy reserves, extend lifespan, and improve reproductive success [9, 10].

Some IAS are highly attractive to mosquitoes as they tend to be more widespread and often have longer active flowering periods, thus guaranteeing a reliable supply of sugar meals and thereby enhancing the mosquito lifespan [11]. A particularly important example is the invasive neotropical weed *P. hysterophorus*, which has become one of Africa’s greatest threats to biodiversity [12]. In addition, feeding on *P. hysterophorus* negatively affects animal health [13], and its pollen and allelopathic substances can cause respiratory problems and skin allergies in humans [13, 14]. *Parthenium hysterophorus* deteriorates soil quality, exacerbates competition for water resources, and may facilitate the dissemination of vector-borne diseases via habitat modification [15], resulting in major ecological and public health issues in impacted areas [16, 17].

Previous studies have shown that female *Anopheles gambiae* Giles (Diptera: Culicidae), the most important malaria vector in sub-Saharan Africa (SSA), is highly attracted to *P. hysterophorus* plants [10]. Feeding on this plant improves its fitness compared with feeding on other, noninvasive plants [15, 18], enhancing its ability to transmit malaria [15, 19]. Moreover, *P. hysterophorus* can also influence the oviposition behavior of mosquito females, as its plant litter can alter the microhabitat conditions, making them more suitable for breeding [18, 20, 21]. Overall, these factors can potentially lead to an increase in mosquito populations in areas infested with *Parthenium*, subsequently affecting the transmission dynamics of mosquito-borne diseases.

Previous investigations on plant feeding have concentrated mainly on *Anopheles gambiae* concerning malaria transmission. Nonetheless, there remains a significant gap in our understanding of how plant feeding, particularly the impact of IAS such as *P. hysterophorus*, affects the transmission dynamics and ecological interactions of mosquito vectors linked to arboviral diseases. These mosquitoes, including species of the genera *Aedes*, *Culex*, and *Mansonia*, play a significant role in the spread of viruses such as dengue, Zika, chikungunya, Rift Valley, and yellow fever viruses [18]. Considering that elevated populations of mosquitoes are typically linked to a rise in pathogen spread, such as arboviruses [22], it is essential to comprehend the relationships between floral resources and these disease-carrying insects. We therefore investigated how floral diversity in Baringo County, Kenya, an arboviral endemic region, affects mosquito proliferation and their plant-feeding behavior, with a particular focus on *P. hysterophorus* and arboviral mosquito vectors.

## Methods

### Study site

**Fig. 1:**
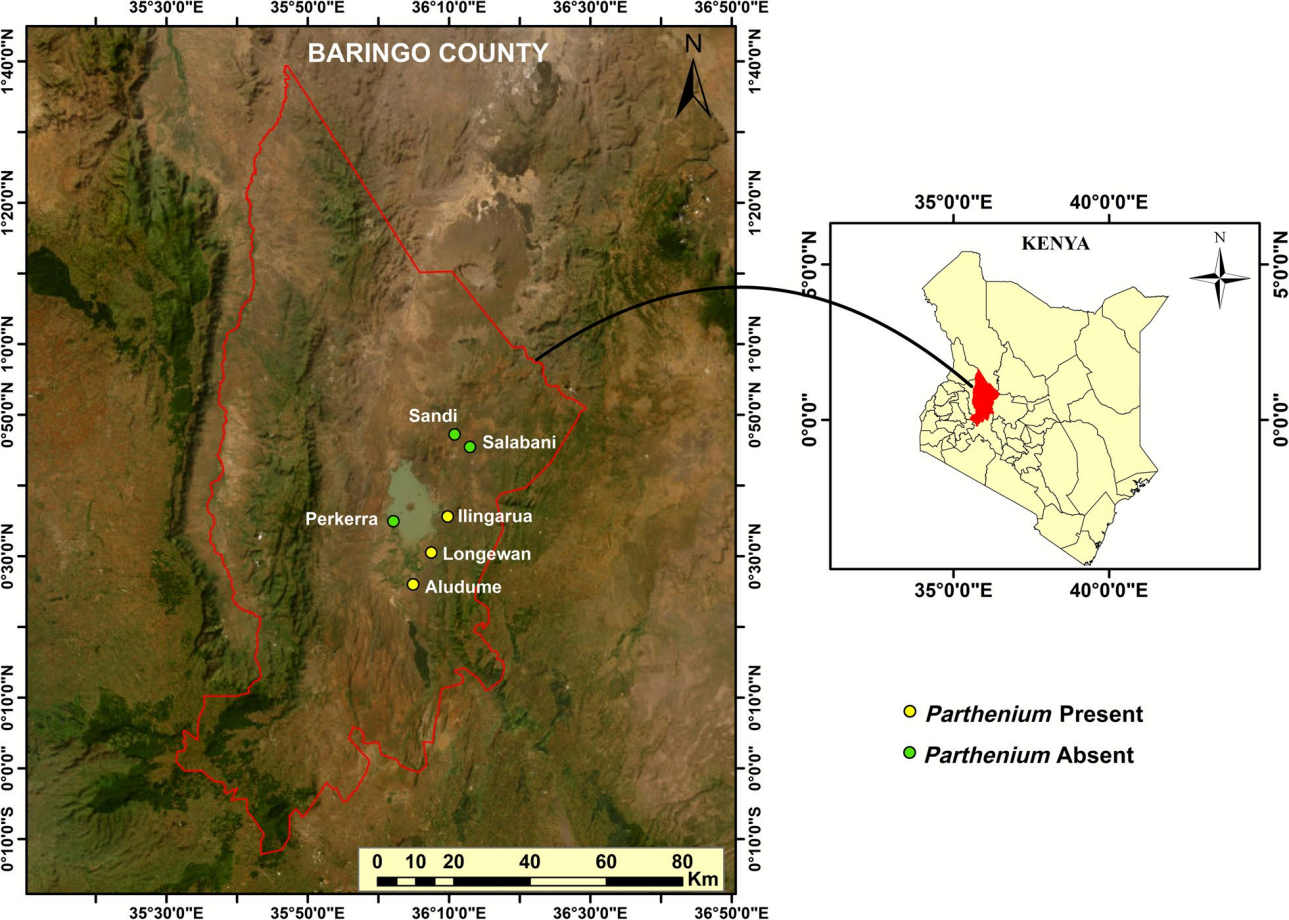
Map of Baringo County, Kenya, showing study sites, which include villages with a known presence of (Aludume, Ilingarua, and Longewan) and those without (Perkerra, Salabani, and Sandai). Map generated in ArcGIS Pro version 3.0 (ESRI, Redlands, CA, USA)

The study was conducted in the Kenyan Rift Valley, specifically in Baringo County (Fig. 1), which lies between latitudes 0° 13′ S and 1° 40′ N and longitudes 35° 36′ and 36° 30′ E. The county is crossed by the Equator. The area experiences annual temperatures ranging between 21 and 32 °C, with precipitation between 300 mm and 1500 mm distributed over two rainy seasons, i.e., March/April to May/June, termed the long rains, and October to early December, termed the short rains [23]. The vegetation is diverse, with key zones that include Acacia–Commiphora bushlands in low-lying semi-arid areas, dominated by *Vachellia* (*Acacia*) *tortilis* (Forssk) Galasso & Banfi (Fabaceae) and *Commiphora africana* (A. Rich) Endl. (Burseraceae). Baringo’s dry montane forests, found in the Tugen Hills, host species such as *Juniperus procera* Hochst ex Endl. (Cupressaceae) [24], while the riverine vegetation along rivers such as the Perkerra includes *Ficus sycomorus* L. (Moraceae) and *V*. (*Acacia*) *xanthophloea* (Benth.) P. J. H. Hurter [25]. Grasslands and shrublands in the lowlands of the county support grasses such as *Chloris gayana* Kunth and *Cynodon dactylon* (L.) Pers. (both Poaceae) [25]. IAS such as *Neltuma* (*Prosopis*) *juliflora* (Sw.) (Fabaceae) [26], and *P. hysterophorus* L. (Asteraceae) have spread in areas such as Marigat, displacing native plants and causing ecological and economic damage [27]. Parts of the county are prone to frequent flooding associated with outbreaks of Rift Valley fever virus, which the area has experienced in the recent past [28–30]. The villages selected for this study included Aludume, Ilngarua, and Longewan, which are infested with *P. hysterophorus*, alongside Perkerra, Salabani, and Sandai, which are generally *P. hysterophorus* free (Fig. 1).

### Study design and sampling

**Fig. 2:**
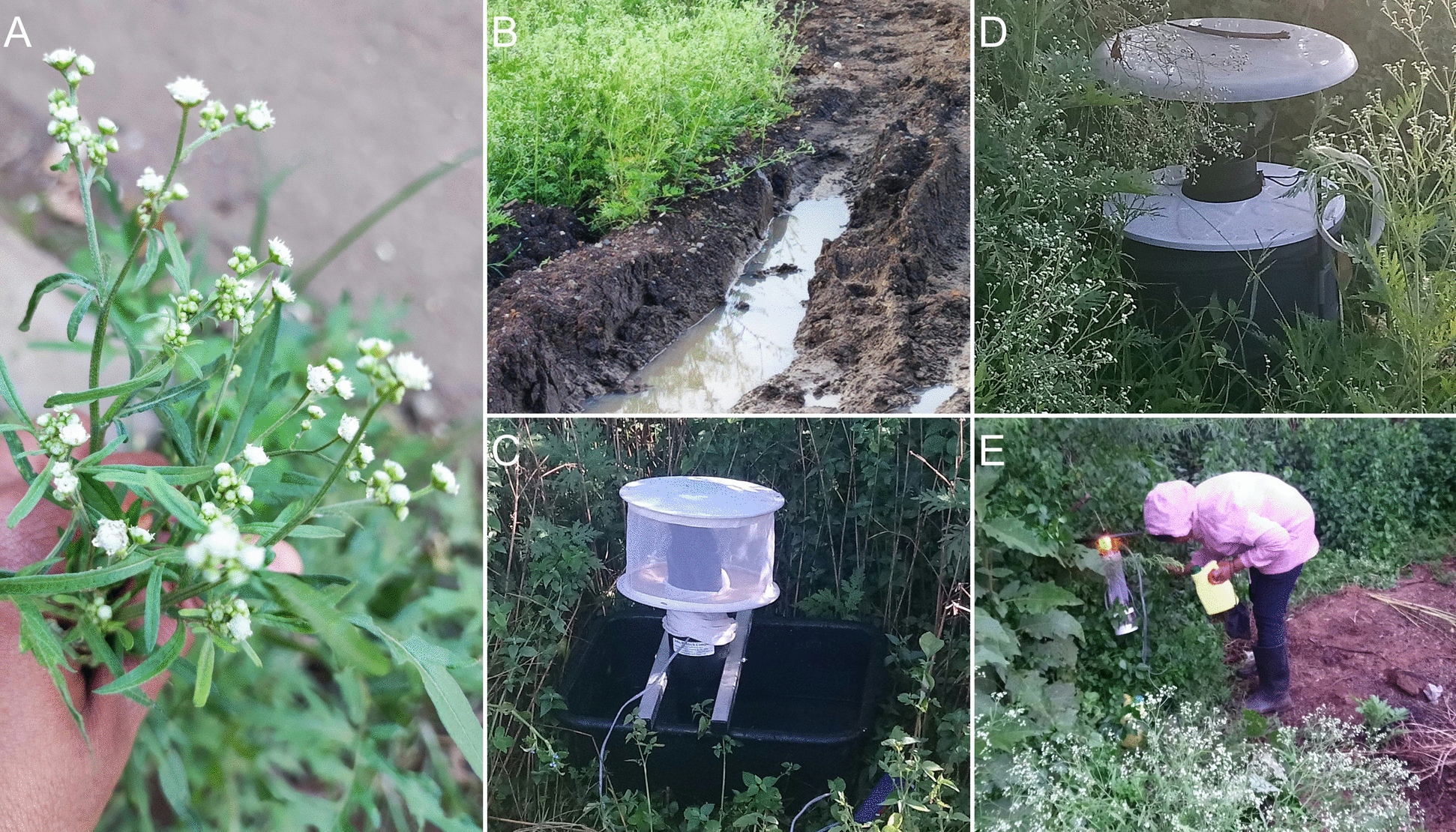
Field setup and sampling activities during mosquito collection in Baringo County, Kenya. **A** The invasive weed *Parthenium hysterophorus* in bloom. **B** *P. hysterophorus*-infested agricultural field, showing typical moist furrows that provide suitable mosquito breeding habitats. **C** BG-Sentinel trap placed near vegetation for capturing day-active mosquitoes. **D** CDC light trap positioned among *P. hysterophorus* vegetation to target nocturnal mosquito species. **E** Field technician inspecting a CDC gravid trap during evening collections

**Fig. 3:**
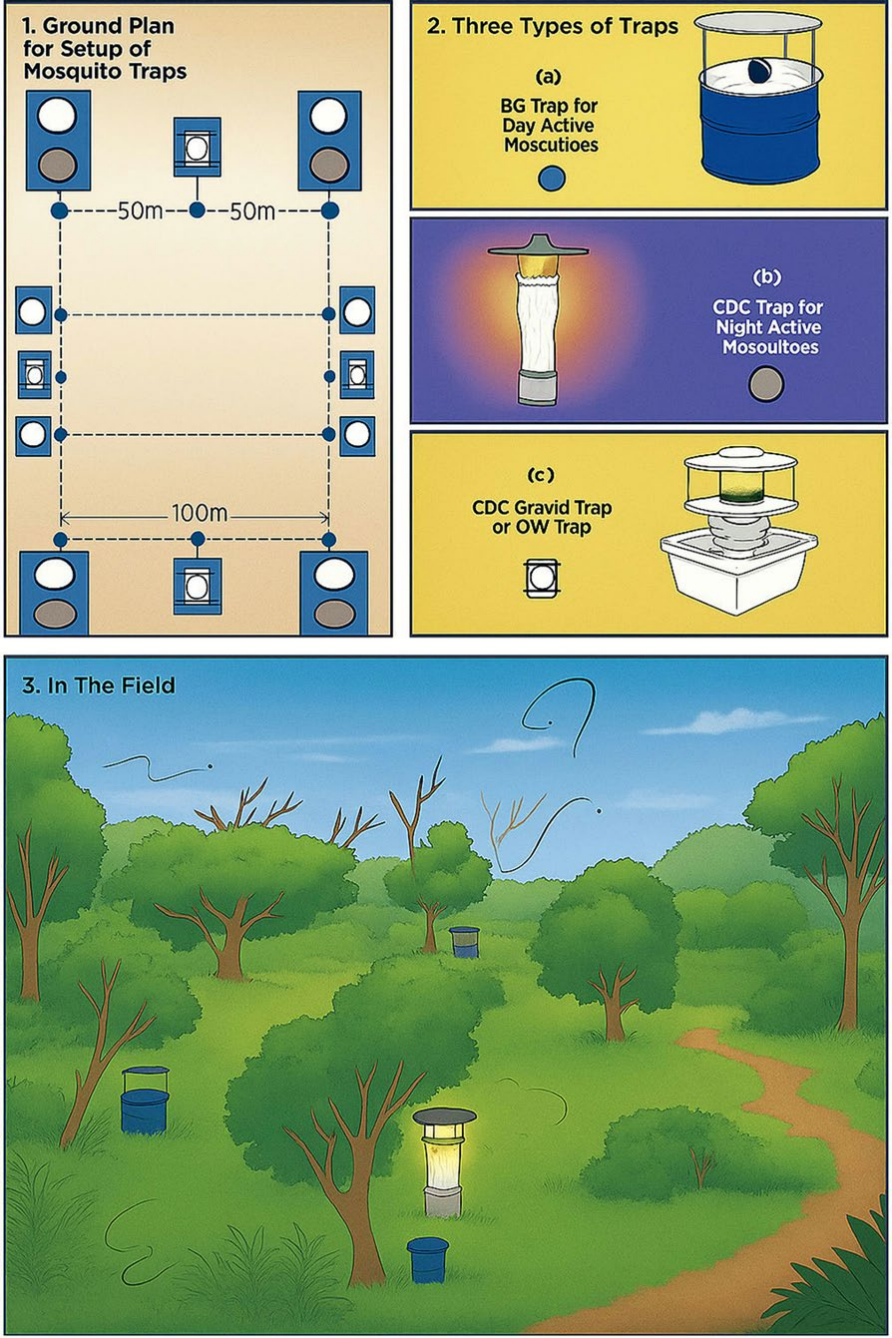
Strategic layout of BG-Sentinel traps, CDC small light traps, and CDC gravid traps in the field to capture mosquitoes of different species and behaviors.

Mosquito sampling was conducted in six villages within Baringo County, Kenya, to assess the influence of *P. hysterophorus* infestation on mosquito abundance and diversity. To enable comparison, the study design comprised three heavily *P. hysterophorus-*infested locations and three ecologically comparable, control sites that were not infested to facilitate robust comparisons. Sampling occurred from October 2019 to March 2020, encompassing both dry and wet seasons to address seasonal variations in mosquito populations. Each site was sampled once during each season, with a duration of five consecutive days per site. This approach ensured that we caught seasonal fluctuations while collecting data consistently across all sites. At each site, three different types of traps were used to capture adult mosquitoes: Biogents (BG)-Sentinel traps, Centers for Disease Control and Prevention (CDC) small light traps, and CDC gravid traps (Figs. 2 and 3). Twenty trap placements were created, including eight BG-Sentinel traps, eight CDC small light traps, and four CDC gravid traps. All traps were positioned close to vegetation and zones of human activities. BG-Sentinel targeted day-active species, such as *Aedes aegypti*, while CDC small light traps, situated at a height of 1.5 m above the ground, were utilized to catch night-active mosquitoes, including *Anopheles* and *Culex* spp. CDC gravid traps, located near water sources, were used to attract gravid mosquitoes.

The traps were supplied with dry ice to release carbon dioxide (CO_2_), acting as a mosquito attractant [31]. To minimize the overlap in mosquito captures, the BG-Sentinel and CDC small light traps were situated 10 m apart at each location. Trap locations were additionally separated by 100 m to reduce the potential interaction between collections.

Moreover, CDC gravid traps were carefully positioned approximately halfway between the BG-Sentinel and CDC small light traps to enhance the probability of capturing gravid mosquitoes. Trapping was performed over five consecutive days at each site. BG-Sentinel traps functioned during daytime hours (06:00–17:00), whereas CDC small light traps and CDC gravid traps were utilized overnight (18:00–06:00). This method facilitated a thorough evaluation of mosquito populations, encompassing individuals with diverse activity patterns and reproductive status. The detailed trapping schedule was the following: 8 BG-Sentinel traps × 5 days = 40 trap-days, 8 CDC mall light traps × 5 days = 40 trap-days, and 4 CDC gravid traps × 5 days = 20 trap-days. Thus, in total, we had 100 trap-days per location.

### Mosquito sample preparation and processing

The samples were delivered to a temporary field laboratory at the Kenya Forestry Research Institute (KEFRI) in Baringo County for processing. Non-mosquito specimens were discarded, while the mosquitoes were initially preserved in liquid nitrogen and then transported in cryovials to the Kenya Medical Research Institute (KEMRI) in Nairobi for morphological identification using taxonomic keys [32, 33]. Mosquitoes were grouped into pools containing no more than 25 individuals according to site, species, sex, trap type, and fed status. Following identification, the mosquitoes were transferred to ILRI, where they were stored at −80 °C for long-term preservation.

### Plant feeding assay

#### Anthrone sugar test

The anthrone test is a biochemical assay to detect the presence of sugar (fructose) [34] and can be an indicator of recent sugar or plant feeding in mosquitoes. Before the test, each mosquito was rinsed in phosphate-buffered saline (PBS) to remove plant residues from their exoskeletons before further analysis. The mosquito’s abdomen was then dissected with a scalpel and macerated in 50 μL absolute ethanol using a sterilized pestle. An aliquot of 25 μL of each homogenate was transferred to the wells of a flat-bottomed 96-well microplate for analysis. The assay is based on the original procedure by Handel and Day [35], with modifications by Matheson et al. [36] to improve the accuracy and sensitivity of detecting fructose. Briefly, 200 μL of anthrone solution (0.15% anthrone (Sigma-Aldrich GmbH, Germany) w/v in 71.7% sulfuric acid) was added to each well containing homogenate. The plate was incubated at room temperature (25 °C) for 60 min. A positive result was indicated by a color change from yellow to blue, indicating the presence of fructose in the sample.

#### DNA extraction

For plant DNA extraction from fructose-positive mosquito samples, the insect homogenate was further homogenized using ceramic beads and a tissue lyser. DNA was then extracted using the ISOLATE II Plant DNA Kit (QIAGEN, Germany) according to the manufacturer’s instructions with modifications. These included the extension of the incubation period with the lysis buffer PA1 and RNase by 4–6 h and elution with Buffer PG for 10 min. The extracted DNA was further analyzed on 1% agarose gel under ultraviolet (UV) light and subsequently quantified by a spectrophotometer NanoDrop™ 2000 (ThermoScientific, USA) and the fluorometer Qubit^®^ 2.0 (Invitrogen, USA). DNA was stored at −20 °C until further analysis, which included polymerase chain reaction (PCR) screening and sequencing.

#### PCR screening and sequencing

PCR was conducted to identify the plant species upon which the mosquitoes had presumably fed, for each pool that tested positive for fructose. Three different sets of primers, i.e., MatK (MatK-413f-1 and MatK-1227r-1), rbcL (rbcL-1F and rbcL-724R), and ITS (ITS1 and ITS4), were used to amplify both conserved rbcL and polymorphic MatK genes of the chloroplast and the 5.8S ITS sequence as described by Kress et al. [37]. These markers are widely recognized in plant barcoding studies [38]. The PCR used MyTaq Polymerase DNA polymerase kit (Bioline, London, UK) in a reaction volume of 10 μL, which contained 10 μM of each forward and reverse primer, 0.0625 U MyTaq DNA polymerase, 5× MyTaq reaction buffer, and 1–2 μL of DNA template (1–10 ng). The reactions were performed in a thermal cycler (Eppendorf Mastercycler Nexus, Germany), following a thermal profile that included an initial denaturation at 94°C for 4 min, followed by 35 cycles of 94°C for 30 s, 52–55 °C for 30 s, and 72 °C for 1 min, concluding with a final extension at 72 °C for 10 min [39]. Molecular-grade water was included as a negative control in all PCRs, while DNA extracted directly from the tissue of selected plant species located within a 100-m radius of each trap site served as a positive control. The PCR products were resolved by 1.5% agarose gel electrophoresis, stained with SYBR Green (Sigma-Aldrich GmbH, Germany), and compared against a 100-bp DNA ladder (HyperLadder, Bioline, London, UK). PCR products were purified and then Sanger sequenced to generate both forward and reverse reads at the BecA-ILRI (The Biosciences eastern and central Africa-International Livestock Research Institute) Segoli laboratory in Nairobi, Kenya. They were visually inspected, aligned, and edited using Geneious Prime version 2023.1 [40]. Each sequence was compared with a reference sequence in the GenBank database using BLASTn (www.ncbi.nlm.nih.gov) and the Barcode of Life Database (BOLD) system database (www.boldsystem.org). Sequences were assigned to specific plant species based on a match threshold of >98%.

### Statistical analysis

Total mosquito species-specific abundance was analyzed using general linear models (GLMs), considering the impact of seasons (dry and rainy) and the presence of *P. hysterophorus* as contributing factors. The Shannon diversity index was calculated to examine mosquito diversity, with analyses conducted using the “vegan” package in R [41]. In this regard, the Shapiro–Wilk test was performed to assess the normality of the data distribution. The extent of plant feeding (fructose-positive samples) was computed as a percentage of the total number of mosquitoes tested. A chi-squared test of independence was utilized to compare the fructose positivity rates between male and female mosquitoes, where *P. hysterophorus* was present or absent and across seasons. A pairwise comparison of proportions was conducted using the pairwise nominal independence test. All statistical tests were performed using R software, with a significance level of *P* < 0.05.

## Results

### Abundance and distribution of mosquitoes

During the sampling period from October 2019 (rainy season) to February 2020 (dry season), a total of 48,489 mosquitoes were captured: 37,181 in the rainy season (October 2019) and 11,272 in the dry season (February 2020) (Table 1). The collected mosquitoes were classified into two subfamilies: Anophelinae, represented by the *Anopheles* genus, and Culicinae, which included the eight genera: *Aedomyia*, *Aedes*, *Coquillettidia*, *Culex*, *Ficalbia*, *Mansonia*, and *Uranotaenia*. In total, 35 mosquito species were morphologically identified. *Mansonia africana* was the predominant species collected (*n* = 31,692/53,258; 59.5%) followed by *Coq. metallicus* (*n* = 4906/53,258; 9.2%) and *Cx. pipiens* (*n* = 4199/53,258; 7.9%) in decreasing order (Table 1).

**Table 1:** Distribution and abundance of mosquito species collected across six locations in Baringo County, Kenya

Species name	Presence of Parthenium			Absence of Parthenium			Total
	Ilngarua	Longewan	Aludume	Perkerra	Salabani	Sandai	
Ae. aegypti^*^	5	21	0	0	14	4	65
Ae. furcifer^*^	0	0	1	1	0	0	1
Ae. hirsutus^*^	2	2	25	86	5	146	243
Ae. mcintoshi^*^	37	79	46	62	0	14	271
*Ae. metallicus*	0	0	0	0	1	0	1
*Ae. simpsoni*	0	0	0	0	3	0	3
*Ae. sudanensis*	0	0	0	1	0	0	1
*Ae. tarsalis*	0	1	1	0	0	0	2
*Ae. tricholabis*	32	19	21	0	10	1	81
*Aedes* spp.^*^	0	4	3	2	3	2	15
*Aedomyia furfurea*	1	0	3	3	1	0	5
*An. coustani*	20	51	17	12	2	3	139
An. funestus^*^	16	14	5	4	6	32	86
An. gambiae^*^	22	9	26	199	23	91	353
*An. malculipalpis*	0	0	0	0	1	0	1
An. nili^*^	0	0	1	0	0	6	6
An. pharoensis^*^	1	1	0	0	0	1	4
*An. squamosus*	0	0	0	0	0	0	0
*Anopheles* spp.^*^	6	0	16	2	1	27	36
*Coq. aurites*	210	36	33	60	42	82	466
*Coq. fuscopennatus*	60	11	12	39	80	25	226
Coq. mettallicus^*^	1734	664	427	341	1005	735	5143
*Coquillettidia* spp.^*^	0	1	0	1	0	0	3
*Cx. annulioris*	122	49	35	86	4	123	433
*Cx. bitaeniorhynchus*	1	5	3	2	0	1	14
Cx. cinereus^*^	4	0	5	4	2	2	12
*Cx. ethiopicus*	14	12	11	5	0	40	83
Cx. pipiens ^*^	332	348	701	904	64	1850	3846
*Cx. poicilipes*	3	7	3	8	0	8	33
Cx. tarsalis^*^	0	2	0	0	0	0	4
*Cx. tigripes*	20	16	110	16	8	68	144
Cx. univittatus ^*^	288	127	573	273	86	796	1697
Cx. vansomereni^*^	2	0	0	3	9	0	14
*Cx. zombaensis*	3	0	0	1	0	0	4
*Culex* spp^*^	60	13	40	35	27	122	267
*Ficalbia mediolineata*	4	7	7	3	0	1	22
*F. splendens*	4	4	3	5	0	2	19
Mansonia africana^*^	23,830	5353	239	1701	397	172	36,806
Mn. uniformis^*^	1376	360	84	328	88	97	2,609
*Mansonia* spp.^*^	69	5	0	7	7	2	95
*Uranotaenia* spp.	0	1	2	0	0	0	2
Total	28,278	7222	2453	4194	1889	4453	48,489

^*^Medically important

#### Effect of seasonal variation and *Parthenium* on mosquito abundance

**Fig. 4:**
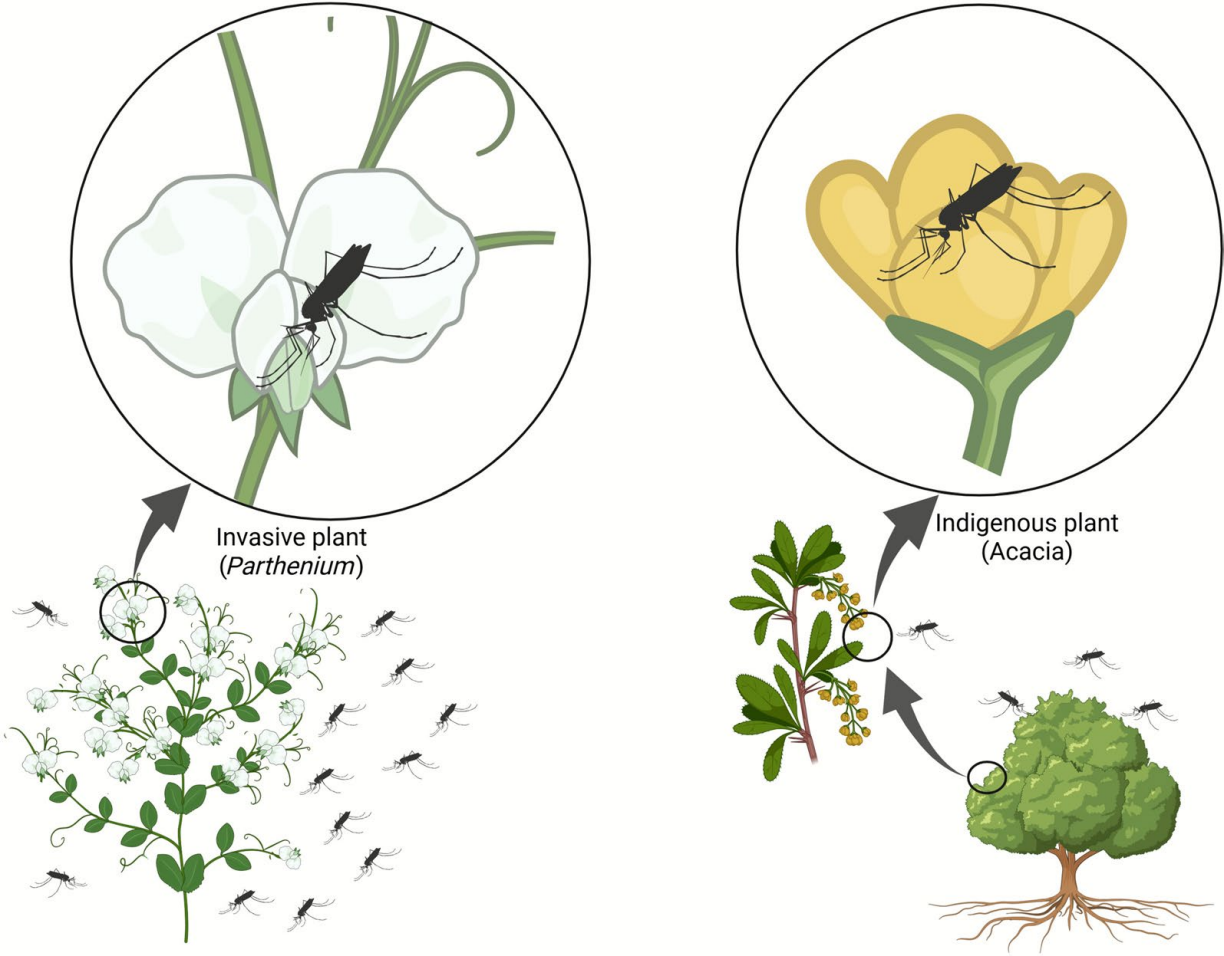
Differential mosquito visits to invasive and native plant species. The graphic illustrates mosquito abundance in relation to the invasive species *Parthenium hysterophorus*, with the native plant *Acacia* spp. An elevated quantity of mosquitoes is recorded engaging with *P. hysterophorus*, indicating its possible function in augmenting mosquito longevity via enhanced sugar availability, with ramifications for vector ecology and disease transmission. Source: authors with BioRender.com (www.biorender.com)

**Fig. 5:**
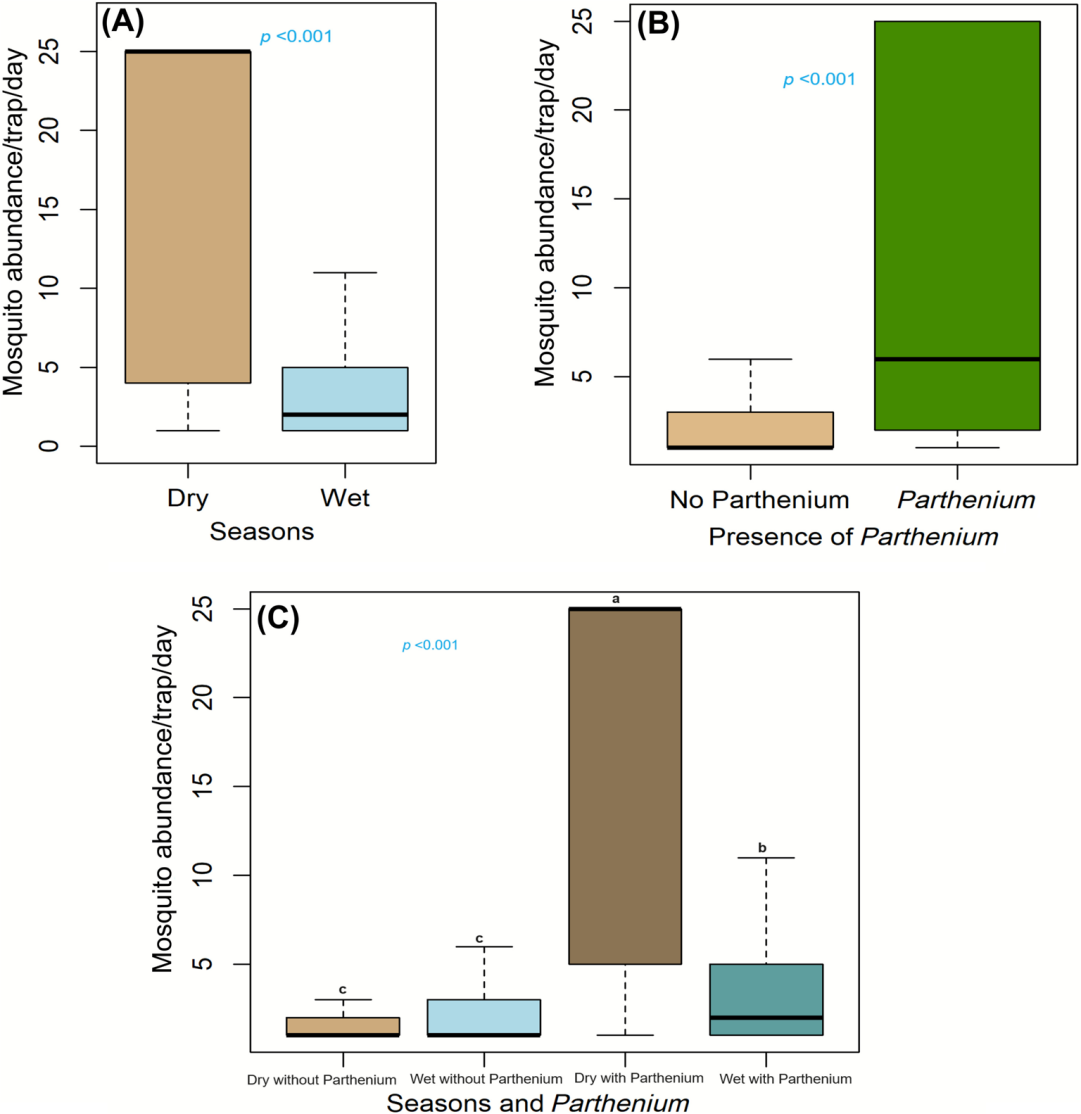
Box plots illustrating the daily mosquito abundance per trap in various environments, during dry and wet seasons (**A**), in the presence and absence of Parthenium (**B**), and accounting for both factors (season and *Parthenium*) (**C**). Asterisks (*) and different letters denote significant differences at *P* < 0.001

The GLM analysis with Poisson distribution revealed a significant seasonal effect, with mosquito abundance being significantly higher during the dry compared with the wet season (*P* < 0.001) (Fig. 5A). The estimated marginal means indicated that the dry season provided more favorable conditions for mosquito proliferation in the study area. The effect of the presence of *P. hysterophorus* on mosquito abundance was also evaluated using a GLM with Poisson distribution (Figs. 4, 5B). The model showed that sites infested with *P. hysterophorus* had significantly higher mosquito abundance than non-infested sites (odds ratio (OR) = 1.8031, *P* < 0.001), suggesting that *P. hysterophorus* creates an environment conducive to higher mosquito densities. Further analysis revealed that the abundance of medically important mosquito species was significantly higher in areas with *P. hysterophorus* (*P* < 0.001). This includes key vectors, particularly *An. gambiae*, *An. funestus*, *Culex pipiens*, *Culex univittatus*, and *Coquillettidia metallicus*.

#### The combined effect of season and presence of *Parthenium*

To investigate the interactive effect of season and presence of *P. hysterophorus* on mosquito abundance, a GLM was fit with an interaction term between season and *P. hysterophorus* presence (Fig. 5C), revealing a significant interaction effect (*P* < 0.001), with the highest mosquito abundance observed during the dry season in *P. hysterophorus*-infested areas. This abundance was significantly higher than in any other combination of season and *P. hysterophorus*. Conversely, the lowest mosquito abundance was recorded during the rainy season in areas without *P. hysterophorus*. Pairwise comparisons, adjusted using Tukey's method, revealed distinct groupings a, b, and c (Fig. 5), indicating significant differences in mosquito abundance between the interaction groups.

### Mosquito species diversity

**Fig. 6:**
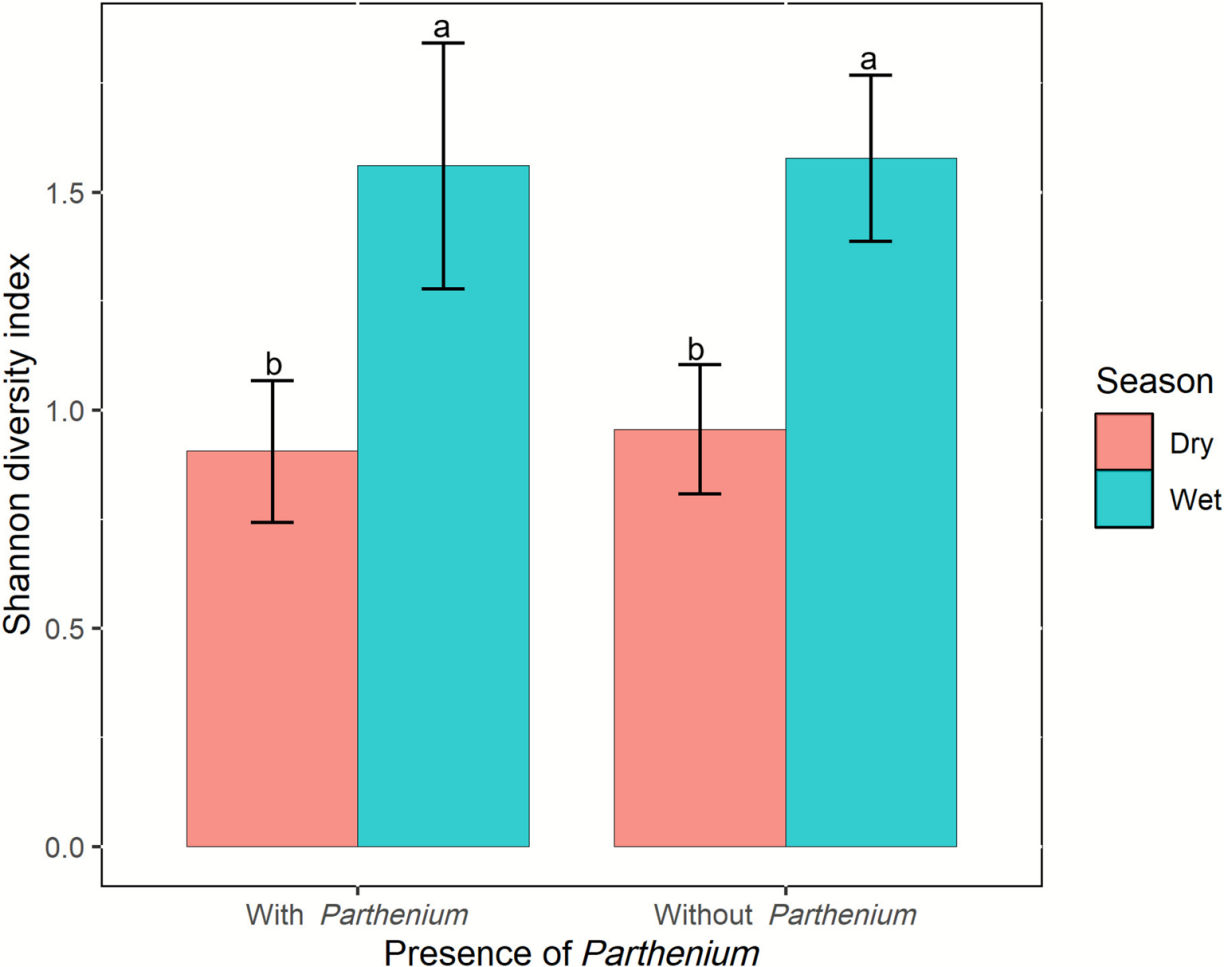
Mean Shannon diversity index (± standard error (SE)) of mosquito populations during the dry and rainy seasons, comparing villages with and without *P. hysterophorus* presence. Different letters indicate significant differences between the seasons in villages with and without *P. hysterophorus* (*t*-test, *P* < 0.05)

In sites free of *P. hysterophorus*, the average Shannon diversity index was higher during the wet (mean = 1.58) compared with the dry season (mean = 0.96) (*t* =  −4.89, *P* = 0.021). Similarly, in sites with *P. hysterophorus*, species diversity was also higher during the wet (mean = 1.56) than the dry season (mean = 0.91) (*t*-value of −2.55, *P*-value = 0.012) (Fig. 6).

### Fructose positivity rates and dynamics of sex ratio

Female mosquitoes had significantly higher fructose positivity rates in areas with *P. hysterophorus* (28.13%; *n* = 299/1,063) compared with areas without *P. hysterophorus* (18%; *n* = 38/209) (*χ*^2^ = 8.3689, *P* = 0.0038), indicating a correlation between the presence of *P. hysterophorus* and increased herbivorous activity in females. The fructose positivity rates for male mosquitoes showed no significant difference between *P. hysterophorus*-infested areas (23.07%; *n* = 27/117) and non-infested areas (15%; *n* = 17/110) (*χ*^2^ = 1.5676, *P* = 0.2106). The chi-squared test revealed no significant difference in overall fructose positivity across sexes in *P. hysterophorus*-infested areas (*χ*^2^ = 1.2122, *P* = 0.2709). Similarly, there was no significant difference between females and males in areas lacking *P. hysterophorus* (*χ*^2^ = 0.20886, *P* = 0.6477). This indicates that the impact of *P. hysterophorus* on fructose positivity was significant solely for female mosquitoes. A thorough chi-squared analysis of all mosquitoes (combined sexes) between *P. hysterophorus*-infested and non-infested regions demonstrated a significant correlation, indicating heightened fructose positivity in *P. hysterophorus*-infested areas (*χ*^2^ = 3.980, *P* = 0.046).

### Diversity of plants identified in sugar-fed mosquitoes

**Fig. 7:**
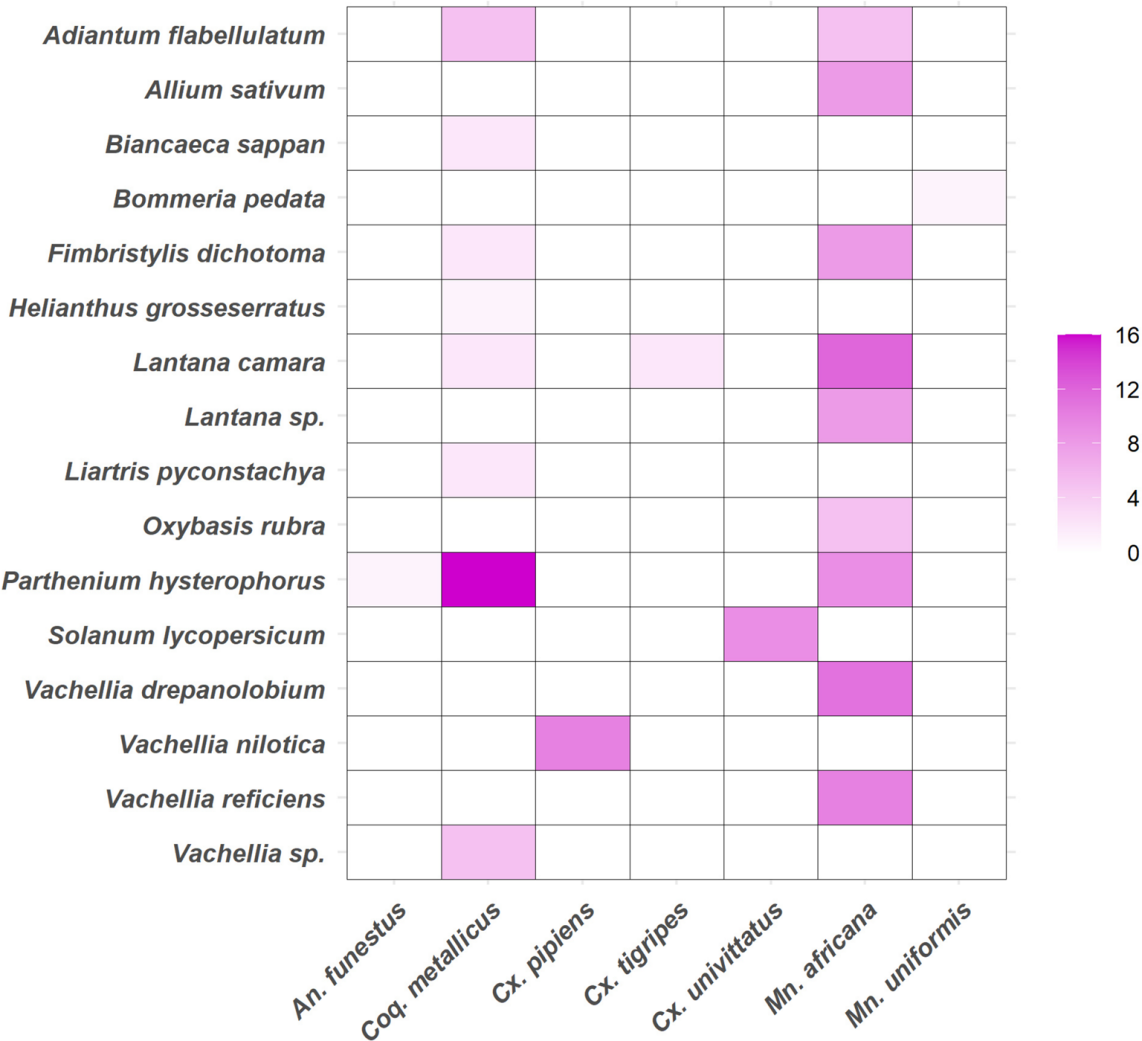
Heatmap displaying plant–host relationships among seven mosquito species from which DNA sequences of fructose-positive samples were successfully amplified. The color intensity in each cell signifies the frequency of identified plant DNA in the corresponding mosquito species, with darker hues representing stronger feeding connections

A total of 476 fructose-positive mosquito samples underwent PCR analysis, providing a 19.7% (94/476) amplification success rate. In total, 47 samples were successfully sequenced from 12 males and 35 females of various mosquito species. The sequenced fragments varied in length from 450 to 660 base pairs. Analysis of the 47 sequences revealed 16 different plant species as potential sources of plant sugars (Fig. 7). The heatmap analysis indicated specific feeding preferences among mosquito species for different plant species. *Mansonia africana* had the most extensive spectrum of plant consumption, with high counts recorded for *Lantana camara* L. (Verbenaceae) (*n* = 12), *V. drepanolobium* (Harms ex. Sjöstedt) P.J.H. Hurter (*n* = 11), and *Vachellia reficiens* (Wawra) Kyal. & Boatwr. (*n* = 10). Importantly, *P. hysterophorus* was recognized as a key plant source for *Mn*. *africana* (*n* = 9). Other plant species found in *Mn. africana* were *Fimbristylis dichotoma* (L.) Vahl (Cyperaceae) (*n* = 8), *Allium sativum* (*n* = 8), and *Lantana sp*. (*n* = 8), while *Oxybasis rubra* (L.) S. Fuentes, Uotila & Borsch (Amaranthaceae) and *Adiantum flabellulatum* (L.) (Pteridaceae) were consumed by *n* = 5 each. In *Coq. metallicus*, notable numbers tested positive for *P. hysterophorus* (*n* = 16), *A. flabellulatum* (*n* = 5), *Vachellia* sp. (*n* = 5), *L. pycnostachya*, *Biancaeca sappan* (L. 1753) Tod. 1875 (Fabaceae), *F. dichotoma*, and *L. camara*, all of which were consumed by two individuals (*n* = 2) each, while *Helianthus grosseserratus* M. Martens (Asteraceae) (*n* = 1) was also detected in the same species. *Anopheles funestus* tested positive only for *P. hysterophorus* (*n* = 1). In *Cx. pipiens*, *V. nilotica* was detected (*n* = 10), while in *Cx. tigripes*, *L. camara* was found (*n* = 2). In *Cx. univittatus*, *S. lycopersicum* (*n* = 9) was found. *Mn. uniformis* tested positive only for *Bommeria pedata* Sw. (Pteridaceae) (*n* = 1) (Fig. 7).

## Discussion

The impact of invasive plant species on mosquito diversity and abundance, as well as on the potential consequences for transmission of vector-borne diseases, has gained increasing attention [8, 42]. We trapped considerably higher numbers of *Mn. africana*, *Coq. metallicus*, and *Cx. pipiens* during the dry season in villages where *P. hysterophorus* was present compared with *Parthenium*-free sites, suggesting a potential positive ecological association between the mosquitoes and the IAS. The high abundance of *Mansonia* spp. is likely driven by the favorable larval habitat provided by wetland vegetation, as these mosquitoes attach to aquatic plants for respiration through modified syphons. Furthermore, *M. africana* exhibited a significant preference for *P. hysterophorus* nectar and elevated fructose positivity, suggesting that the proliferation of the species in infested areas is significantly influenced by the availability of floral sugar and the suitability of the larval habitat [43, 44]. However, additional factors certainly contributed to the elevated mosquito populations. These may include humans and livestock as suppliers of blood and water storage containers as breeding locations. Mosquitoes may also receive sugar from other flowering plants [10]. The epidemiological importance of these mosquito species is highlighted by their roles as vectors for several arboviruses. *Mansonia*, *Coquillettidia*, and *Culex* spp. transmit, among others, the Rift Valley fever virus (RVFV), which can affect both humans and livestock [45, 46]. Additionally, *Cx. pipiens* is a key vector for the West Nile virus, lymphatic filariasis, Rift Valley fever virus, and encephalitic viruses, which have serious implications for human health [47, 48]. The high prevalence of these vector species in Baringo County emphasizes their potential influence on public health, warranting continuous surveillance and vector control measures. Plant sugar assays and DNA barcoding also showed that most of the mosquitoes were feeding on *P. hysterophorus* and *L. camara*, suggesting that these plants potentially serve as a crucial source of sugar for mosquitoes.

Our findings indicate a complex and species-specific relationship between the presence of *P. hysterophorus* and the abundance of mosquitoes. Interestingly, *An. gambiae* a key malaria vector, was found to be more prevalent in sites devoid of *Parthenium* (Table 1), contradicting previous studies that identified *P. hysterophorus* as an essential sugar source boosting the survival and activity of *An. gambiae* [10, 49]. The prior findings, mainly derived from controlled experiments, indicated a significant preference for *P. hysterophorus* over other plant species such as *Senna occidentalis* (L..) Link (Fabaceae) [10, 50, 51]. Conversely, our field data reveal that, in natural settings, *An. gambiae* populations might be more affected by various ecological factors, including host availability, microhabitat traits, or alternative sugar sources. Our research highlights that field conditions may influence mosquito–plant interactions differently from laboratory settings.

Other IAS have also been found to enhance the abundance of mosquitoes; for example, in Mali, a drastic drop in *Anopheles* spp. populations and sugar feeding was observed in areas where the flowering parts of *Neltuma* (*Prosopis*) *juliflora* (Sw.) Raf. (Fabaceae) were removed compared with areas with intact stalks. Notably, most of these studies focused on *Anopheles* mosquitoes; hence, our study is crucial in providing information on plant foraging in arboviral mosquito vectors such as *Mansonia* and *Culex* spp. In this regard, *Aedes aegypti* mosquitoes were found to be frequently feeding on Fabaceae plants in the coastal town of Kilifi; however, some fed on *P. hysterophorus* [52]. Typically, mosquito abundance is higher during the rainy season because of the greater availability of breeding habitats and favorable humidity and temperature [53, 54]. Yet, we found the exact opposite in areas where *Parthenium* was present, with significantly higher mosquito trap catches during the dry than the wet season, something that we did not observe in areas where the IAS was absent. One of the contributing factors could have been that farmers in Baringo County do not control weeds during the dry season, thereby allowing *P. hysterophorus* in affected areas to flourish. In contrast, during the rainy (growing) season, fields are cleared for crop cultivation, resulting in the removal of *Parthenium*. Hence, the abundance of *P. hysterophorus* during the dry season in affected areas is likely to provide favorable microhabitats and sugar-rich food sources for mosquitoes, thereby enabling their survival and reproduction even when traditional breeding sites are in short supply [27, 42, 55]. *Parthenium hysterophorus* thus helps to sustain mosquito populations during periods of low to no precipitation, highlighting its potential to affect mosquito dynamics at the ecological level.

In our study, we found *P. hysterophorus* to be considerably more preferred by mosquitoes than other invasive plant species such as *L. camara* and also *Vachellia reficiens* (Wawra) Kyal. & Boatwr. (Fabaceae). Mosquitoes demonstrate selective plant-feeding preferences, and while studies show that *P. hysterophorus* has poor nutritional content, it is likely to attract mosquitoes through release of volatile compounds [15, 17, 56]. It is also likely to outcompete native flora, especially during the dry season, resulting in a reduction in the availability of native nectar sources and compelling mosquitoes to adapt their feeding habits [57, 58]. Mosquitoes that frequently fed on *P. hysterophorus* included *Coq. metallicus*, *Mn. africana*, and *An. funestus*. *Mansonia* spp. are recognized vectors of RVFV and lymphatic filariasis [59] (and in the last major RVF outbreak in Kenya, the virus was frequently detected in *Mn. africana* and *Mn. uniformis* mosquitoes in Baringo County [30]. *Anopheles funestus* is one of the principal malaria vectors in SSA [60]. Their high trap catches in areas infested with *P. hysterophorus* suggest that the IAS may elevate the risk of arboviral disease transmission.

We found a significant correlation between the presence of *P. hysterophorus* and elevated fructose positivity in female mosquitoes, suggesting that the IAS acts as a crucial sugar source. Nyasembe et al. [15] observed that *P. hysterophorus* enhances *An. gambiae* longevity by providing ample sugar sources, and *P. hysterophorus* was favored above other invasive species such as *L. camara*, indicating its distinct appeal. While the presence of IAS such as *P. hysterophorus* can potentially increase disease risk by increasing mosquito abundance, other studies have shown that sugar intake prior to blood feeding may protect *Ae. aegypti* females against infection with Semiliki Forest and Zika viruses [61]. On the basis of previous studies, this effect can also be extrapolated to *Anopheles* and *Culex* spp. mosquitoes [62, 63] The protective effect is thought to be mediated through increased melanization and stimulation of the mosquito immune system [61]. This suggests that plant sugar in mosquitoes could reduce their capacity to harbor and transmit viruses. Further field and in vitro experiments are therefore required to disentangle these contradictory dynamics in plant feeding and arboviral transmission.

## Conclusions

Our study indicates a distinct association between the presence of *P. hysterophorus* and increased mosquito populations, particularly under unfavorable dry conditions, stressing the crucial role of IAS-like *P. hysterophorus* as both a sugar source and possible breeding microhabitat for mosquitoes, enabling vector persistence during adverse environmental conditions. Furthermore, temperature, humidity, and land-use patterns can shape mosquito populations by driving resource availability and the suitability of breeding sites. DNA barcoding and sugar analyses identified *P. hysterophorus* as a significant sugar source for various medically important mosquito species, including *Mn. africana* and *Cx. pipiens*. Importantly, mosquito diversity remained consistent throughout the year in *P. hysterophorus*-infested areas, in contrast to control areas that displayed seasonal variations. These findings indicate that the effective management of *P. hysterophorus* could function as a strategic approach for controlling mosquito proliferation, thereby reducing the risk and spread of vector-borne diseases.

## Data Availability

Data supporting the main conclusions of this study are included in the manuscript.

## References

[1] International Union for Conservation of Nature (IUCN). IUCN global standard for nature-based solutions: a user-friendly framework for the verification, design and scaling up of NbS. 1st ed. Gland: IUCN; 2020. 10.2305/IUCN.CH.2020.08.en.

[2] Hollis L. Weeding invasive alien species—Africa’s economic burden. 2021 [Internet]. [cited 2025 Jul 1]. https://blog.invasive-species.org/2021/05/25/weeding-invasive-alien-species-africas-economic-burden/

[3] Duguma G, Fitamo D, Kebede F. Socioeconomic and ecological consequences of *Parthenium* weed (*Parthenium hysterophorus* L.) in Boset Woreda, Ethiopia. Afr J Agric Res. 2019;14:1921–42.

[4] Seebens H, Blackburn TM, Dyer EE, Genovesi P, Hulme PE, Jeschke JM, et al. No saturation in the accumulation of alien species worldwide. Nat Commun. 2017;8:14435. 10.1038/ncomms14435. 28198420 10.1038/ncomms14435 PMC5316856

[5] Kaur M, Aggarwal NK, Kumar V, Dhiman R. Effects and management of *Parthenium hysterophorus*: a weed of global significance. Int Sch Res Notices. 2014;2014:368647. 27355059 10.1155/2014/368647 PMC4897546

[6] Attardo GM, Hansen IA, Raikhel AS. Nutritional regulation of vitellogenesis in mosquitoes: implications for anautogeny. Insect Biochem Mol Biol. 2005;35:661–75. 15894184 10.1016/j.ibmb.2005.02.013

[7] Takala S, Branch O, Escalante AA, Kariuki S, Wootton J, Lal AA. Evidence for intragenic recombination in *Plasmodium falciparum*: identification of a novel allele family in block 2 of merozoite surface protein-1. Mol Biochem Parasitol. 2002;125:163–71. 12467983 10.1016/s0166-6851(02)00237-2 PMC1853304

[8] Stone WJR, Eldering M, van Gemert GJ, Lanke KHW, Grignard L, van de Vegte-Bolmer MG, et al. The relevance and applicability of oocyst prevalence as a read-out for mosquito feeding assays. Sci Rep. 2013;3:3418. 10.1038/srep03418. 24301557 10.1038/srep03418 PMC4894383

[9] Hancock RG, Foster WA. Larval and adult nutrition effects on blood/nectar choice of *Culex nigripalpus* mosquitoes. Med Vet Entomol. 1997;11:112–22. 10.1111/j.1365-2915.1997.tb00299.x. 9226638 10.1111/j.1365-2915.1997.tb00299.x

[10] Manda H, Gouagna LC, Nyandat E, Kabiru EW, Jackson RR, Foster WA, et al. Discriminative feeding behaviour of *Anopheles gambiae* s.s. on endemic plants in western Kenya. Med Vet Entomol. 2007;21:103–11. 10.1111/j.1365-2915.2007.00672.x. 17373953 10.1111/j.1365-2915.2007.00672.x PMC2705332

[11] Muller GC, Junnila A, Traore MM, Traore SF, Doumbia S, Sissoko F, et al. The invasive shrub *Prosopis juliflora* enhances the malaria parasite transmission capacity of *Anopheles* mosquitoes: a habitat manipulation experiment. Malar J. 2017;16:237. 10.1186/s12936-017-1878-9. 28676093 10.1186/s12936-017-1878-9 PMC5497341

[12] Adkins S, Shabbir A. Biology, ecology and management of the invasive *Parthenium* weed (*Parthenium hysterophorus* L.). Pest Manag Sci. 2014;70:1023–9. 24430973 10.1002/ps.3708

[13] Bashar HMK, Juraimi AS, Ahmad-Hamdani MS, Uddin MK, Asib N, Anwar MP, et al. A mystic weed, *Parthenium hysterophorus*: threats, potentials and management. Agronomy. 2021;11:1514. 10.3390/agronomy11081514.

[14] Dheer V, Singh KK, Vaish P, Kumar K, Kumar Y, Singh M, et al. *Parthenium hysterophorus* L.: an overview of management and beneficial aspects. Int J Environ Clim Change. 2023;13:1221–39.

[15] Nyasembe VO, Cheseto X, Kaplan F, Foster WA, Teal PEA, Tumlinson JH, et al. The invasive American weed *Parthenium hysterophorus* can negatively impact malaria control in Africa. PLoS ONE. 2015;10:e0137836. 26367123 10.1371/journal.pone.0137836 PMC4569267

[16] Mao R, Bajwa AA, Adkins S. A superweed in the making: adaptations of *Parthenium hysterophorus* to a changing climate – a review. Agron Sustain Dev. 2021;41:47. 10.1007/s13593-021-00699-8.

[17] McConnachie AJ, Strathie LW, Mersie W, Gebrehiwot L, Zewdie K, Abdurehim A, et al. Current and potential geographical distribution of the invasive plant *Parthenium hysterophorus* (Asteraceae) in eastern and southern Africa. Weed Res. 2011;51:71–84. 10.1111/j.1365-3180.2010.00820.x.

[18] Agha SB, Alvarez M, Becker M, Fèvre EM, Junglen S, Borgemeister C. Invasive alien plants in Africa and the potential emergence of mosquito-borne arboviral diseases—a review and research outlook. Viruses. 2020;13:32. 10.3390/v13010032. 33375455 10.3390/v13010032 PMC7823977

[19] Reisen WK. Landscape epidemiology of vector-borne diseases. Annu Rev Entomol. 2010;55:461–83. 10.1146/annurev-ento-112408-085419. 19737082 10.1146/annurev-ento-112408-085419

[20] David JP, Rey D, Pautou MP, Meyran JC. Differential toxicity of leaf litter to dipteran larvae of mosquito developmental sites. J Invertebr Pathol. 2000;75:9–18. 10.1006/jipa.1999.4886. 10631052 10.1006/jipa.1999.4886

[21] Okazawa T. Effects of blood sources on fertility of a malaria vector, *Anopheles farauti,* in Solomon Islands. Med Entomol Zool. 2001;52:249–52. 10.7601/mez.52.249.

[22] Denóbile C, de Chiba Castro WA, Silva Matos DMD. Public health implications of invasive plants: a scientometric study. Plants. 2023;12:661. 10.3390/plants12030661. 36771745 10.3390/plants12030661 PMC9921203

[23] Kipruto EK, Ochieng AO, Anyona DN, Mbalanya M, Mutua EN, Onguru D, et al. Effect of climatic variability on malaria trends in Baringo County, Kenya. Malar J. 2017;16:220. 10.1186/s12936-017-1848-2. 28545590 10.1186/s12936-017-1848-2 PMC5445289

[24] Maundu P, Tengnäs B. Useful trees and shrubs for Kenya. Nairobi (Kenya): World Agroforestry Centre (ICRAF); 2005. (ICRAF Technical Handbook No. 35).

[25] Beentje HJ. Kenya: trees, shrubs and lianas. Nairobi: National Museums of Kenya; 1994.

[26] Pasiecznik NM, Felker P, Harris PJC, Harsh LN, Cruz G, Tewari JC, et al. The Prosopis juliflora–Prosopis pallida complex: a monograph. Coventry: HDRA; 2001.

[27] Sharma GP, Raghubanshi AS, Singh JS. Lantana invasion: an overview. Weed Biol Manag. 2005;5:157–65.

[28] Anyamba A, Linthicum KJ, Small J, Britch SC, Pak E, de La Rocque S, et al. Prediction and assessment of Rift Valley fever activity in East and Southern Africa, 2006–2008, and possible vector control strategies. Am J Trop Med Hyg. 2010;83:43–51. 20682905 10.4269/ajtmh.2010.09-0289 PMC2913499

[29] Nguku PM, Sharif S, Mutonga D, Amwayi S, Omolo J, Mohammed O, et al. An investigation of a major outbreak of Rift Valley fever in Kenya, 2006–2007. Am J Trop Med Hyg. 2010;83:05–13. 10.4269/ajtmh.2010.09-0288 PMC2913496 20682900

[30] Sang R, Lutomiah J, Said M, Makio A, Koka H, Koskei E, et al. Effects of irrigation and rainfall on the population dynamics of Rift Valley fever and other arbovirus mosquito vectors in the epidemic-prone Tana River County, Kenya. J Med Entomol. 2017;54:460–70. 28011732 10.1093/jme/tjw206 PMC5850818

[31] Silver JB. Mosquito ecology: field sampling methods. 3rd ed. Dordrecht (Netherlands): Springer; 2007.

[32] Edwards FW. Mosquitoes of the Ethiopian region. Part II: culicine adults and pupae. London: British Museum (Natural History); 1941.

[33] Gillies MT, De Meillon B. The anophelinae of Africa south of the Sahara (Ethiopian zoogeographical region). Johannesburg: South African Institute for Medical Research; 1968.

[34] DuBois M, Gilles KA, Hamilton JK, Rebers PT, Smith F. Colorimetric method for determination of sugars and related substances. Anal Chem. 1956;28:350–6.

[35] Van Handel E, Day J. Assay of lipids, glycogen and sugars in individual mosquitoes: correlations with wing length in field-collected *Aedes vexans*. J Am Mosq Control Assoc. 1988;4:241–6. 3225576

[36] Matheson CD, Muller G, Junnila A, Vernon K, Hausmann A, Miller M, et al. A PCR method for detection of plant meals from the guts of insects. Org Divers Evol. 2008;7:294–303.

[37] Kress WJ, Wurdack KJ, Zimmer EA, Weigt LA, Janzen DH. Use of DNA barcodes to identify flowering plants. Proc Natl Acad Sci U S A. 2005;102:8369–74. 15928076 10.1073/pnas.0503123102 PMC1142120

[38] Hollingsworth PM, Li DZ, Van der Bank M, Twyford AD. Telling plant species apart with DNA: from barcodes to genomes. Philos Trans R Soc Lond B Biol Sci. 2016;371:20150338. 27481790 10.1098/rstb.2015.0338 PMC4971190

[39] Forrest L, Hart M. DNA barcoding standard operating protocol: plants and lichens at RBGE, lab methods: PCR and sequencing v1. 2023 [Internet]. [cited 2025 Oct 3]. 10.17504/protocols.io.bp2l697qrlqe/v1

[40] Kearse M, Moir R, Wilson A, Stones-Havas S, Cheung M, Sturrock S, et al. Geneious basic: an integrated and extendable desktop software platform for the organization and analysis of sequence data. Bioinformatics. 2012;28:1647–9. 22543367 10.1093/bioinformatics/bts199 PMC3371832

[41] R Core Team. R: a language and environment for statistical computing. Vienna: R Foundation for Statistical Computing; 2023.

[42] Muturi EJ, Spencer JL, Allan BF. Influence of biofuel crops on mosquito production and oviposition site selection. GCB Bioenergy. 2014;6:61–6. 10.1111/gcbb.12038.

[43] Krishnamoorthy K, Rajendran G, Panicker K. Aquatic vegetation and their natural hospitability to the immatures of *Mansonia* mosquitoes, the vectors of *Brugia malayi* in Shertallai, Kerala, India. Southeast Asian J Trop Med Public Health. 1994;25:760–5. 7667728

[44] Soares Gil LH, Mello CF, Silva JDS, Oliveira JDS, Freitas Silva SO, Rodríguez-Planes L, et al. Evaluation of *Mansonia* spp. infestation on aquatic plants in lentic and lotic environments of the Madeira River Basin in Porto Velho, Rondônia, Brazil. J Am Mosq Control Assoc. 2021;37:143–51. 10.2987/21-7007.1. 34407173 10.2987/21-7007.1

[45] Bird BH, Ksiazek TG, Nichol ST, MacLachlan NJ. Rift Valley fever virus. J Am Vet Med Assoc. 2009;234:883–93. 10.2460/javma.234.7.883. 19335238 10.2460/javma.234.7.883

[46] Linthicum KJ, Britch SC, Anyamba A. Rift Valley fever: an emerging mosquito-borne disease. Annu Rev Entomol. 2016;61:395–415. 10.1146/annurev-ento-010715-023819. 26982443 10.1146/annurev-ento-010715-023819

[47] Farajollahi A, Fonseca DM, Kramer LD, Kilpatrick AM. “Bird-biting” mosquitoes and human disease: a review of the role of *Culex pipiens* complex mosquitoes in epidemiology. Infect Genet Evol. 2011;11:1577–85. 10.1016/j.meegid.2011.08.013. 21875691 10.1016/j.meegid.2011.08.013 PMC3190018

[48] Hayes EB, Komar N, Nasci RS, Montgomery SP, O’Leary DR, Campbell GL. Epidemiology and transmission dynamics of West Nile virus disease. Emerg Infect Dis. 2005;11:1167–73. 10.3201/eid1108.050289. 16102302 10.3201/eid1108.050289a PMC3320478

[49] Ebrahimi B, Jackson BT, Guseman JL, Przybylowicz CM, Stone CM, Foster WA. Alteration of plant species assemblages can decrease the transmission potential of malaria mosquitoes. J Appl Ecol. 2018;55:841–51. 10.1111/1365-2664.13001. 29551835 10.1111/1365-2664.13001 PMC5849257

[50] Gary RE, Cannon JW, Foster WA. Effect of sugar on male *Anopheles gambiae* mating performance, as modified by temperature, space and body size. Parasit Vectors. 2009;2:19. 10.1186/1756-3305-2-19. 19386114 10.1186/1756-3305-2-19 PMC2681455

[51] Kinya F, Milugo TK, Mutero CM, Wondji CS, Torto B, Tchouassi DP. Insights into malaria vectors–plant interaction in a dryland ecosystem. Sci Rep. 2024;14:20625. 10.1038/s41598-024-71205-9. 39232051 10.1038/s41598-024-71205-9 PMC11375087

[52] Wanjiku C, Tchouassi DP, Sole CL, Pirk C, Torto B. Plant sugar feeding patterns of wild-caught *Aedes aegypti* from dengue endemic and non-endemic areas of Kenya. Med Vet Entomol. 2021;35:417–25. 10.1111/mve.12514. 33682949 10.1111/mve.12514

[53] Harrington LC, Edman JD, Scott TW. Why do female *Aedes aegypti* (Diptera: Culicidae) feed preferentially and frequently on human blood? J Med Entomol. 2001;38:411–22. 11372967 10.1603/0022-2585-38.3.411

[54] McIntyre KM, Setzkorn C, Hepworth PJ, Morand S, Morse AP, Baylis M. Systematic assessment of the climate sensitivity of important human and domestic animal pathogens in Europe. Sci Rep. 2017;7:7134. 28769039 10.1038/s41598-017-06948-9 PMC5541049

[55] Mwangi E, Swallow B. *Prosopis juliflora* invasion and rural livelihoods in the Lake Baringo area of Kenya. Conserv Soc. 2008;6:130–40.

[56] Branham EJ, Nelson IE, Rochlin I, Widmer TD, Byers NM, Müller GC, et al. Fantastic feasts and where to find them: mosquito (Diptera: Culicidae) sugar feeding and survivorship on endemic flowers of arid scrublands. Arthropod Plant Interact. 2025;19:45. 10.1007/s11829-025-10151-3.

[57] Hejda M, Pyšek P, Jarošík V. Impact of invasive plants on the species richness, diversity and composition of invaded communities. J Ecol. 2009;97:393–403. 10.1111/j.1365-2745.2009.01480.x.

[58] Levine JM, Vilà M, D’Antonio CM, Dukes JS, Grigulis K, Lavorel S. Mechanisms underlying the impacts of exotic plant invasions. Proc R Soc Lond B Biol Sci. 2003;270:775–81. 10.1098/rspb.2003.2327. 10.1098/rspb.2003.2327 PMC1691311 12737654

[59] Ochuka M, Ikporukpo C, Mijinyawa Y, Ogendi G. Land use/land cover dynamics and anthropogenic driving factors in Lake Baringo catchment, Rift Valley, Kenya. Nat Resour. 2019;10:367. 10.4236/nr.2019.1010025.

[60] Coetzee M, Koekemoer LL. Molecular systematics and insecticide resistance in the major African malaria vector *Anopheles funestus*. Annu Rev Entomol. 2013;58:393–412. 23317045 10.1146/annurev-ento-120811-153628

[61] Almire F, Terry S, McFarlane M, Sziemel AM, Terhzaz S, Varjak M, et al. Sugar feeding enhances gut immunity and protects against arboviral infection in the mosquito vector *Aedes aegypti.* 2021 [Preprint]. 10.1101/2021.01.05.425375 10.1371/journal.ppat.1009870 PMC8412342 34473801

[62] Ferguson HM, Dornhaus A, Beeche A, Borgemeister C, Gottlieb M, Mulla MS, et al. Ecology: a prerequisite for malaria elimination and eradication. PLoS Med. 2010;7:e1000312. 10.1371/journal.pmed.1000312. 20689800 10.1371/journal.pmed.1000303 PMC2914634

[63] Koella JC, Sørensen FL. Effect of adult nutrition on the melanization immune response of the malaria vector *Anopheles stephensi*. Med Vet Entomol. 2002;16:316–20. 12243233 10.1046/j.1365-2915.2002.00381.x

